# Last Place of Care and End-of-Life Quality of Life in the United States: Evidence From a National Representative Data Set

**DOI:** 10.1093/geroni/igaa057.056

**Published:** 2020-12-16

**Authors:** Yifan Lou, Nan Jiang, Katherine Ornstein

**Affiliations:** 1 Columbia University, New York, New York, United States; 2 National University of Singapore, Singapore, New York, Singapore; 3 Icahn School of Medicine at Mount Sinai, New York, New York, United States

## Abstract

Background: Quality of life (QoL) during last stage of life has raised expanded interests as an important aspect of person-centered care. Last place of care (LPC), refer to the last place decedents received their formal end-of-life care (EOLC), has been identified as a key indicator of older adults’ end-of-life QoL, but the relationship was understudied. This study explores the association between LPC and end-of-life QoL among American older adults. Methods: Data used seven waves of Last Month of Life data with a total sample of 3068 Medicare decedents in NHATS. Outcome is end-of-life QoL assessed by eleven measures on four domains: pain and symptoms management (SP), quality of healthcare encounter (HE), person-centered care (PC), and overall quality of care (QC). LPC was categorized into home, hospital, nursing home, and residential hospice. Multivariate logistic regression analyses were used to examine the relationship with covariates. Results: LPC varied by most demographic characteristics, except immigration status and education. Older adults whose LPC is hospital, compared to those who had home-care, were less likely to have great experiences on HE, PC, and QC. People dying at nursing homes are more likely to receive care meeting their dyspnea and spiritual needs. Residential hospice is negatively related to respected care, clear coordination, and keeping family informed, but are more likely to provide PS and spiritual care. Discussion: Home-based end-of-life care has certain advantages but still has room to improve on SP and religious concerns. Hospitals should keep reforming their service delivery structure to improve patients’ QoL.

